# Multi‐omics analysis of neuron‐derived small extracellular vesicles to identify novel peripheral biomarker of cognitive impairment in individuals with type 2 diabetes

**DOI:** 10.1002/alz.091878

**Published:** 2025-01-09

**Authors:** Ashish Kumar, Jacob Alexander Cleary, Yixin Su, Sangeeta Singh, Marjorie Howard, Kathleen M. Hayden, Jingyun Lee, Cristina M Furdui, Mark A. Espeland, Jingzhong Ding, Gagan Deep

**Affiliations:** ^1^ Wake Forest University School of Medicine, Winston Salem, NC USA; ^2^ Wake Forest School of Medicine, Winston‐Salem, NC USA; ^3^ Wake Forest University School of Medicine, Winston‐Salem, NC USA

## Abstract

**Background:**

Central nervous system (CNS) dysregulated insulin and peripheral hyperinsulinemia has been associated with AD. However, analyzing CNS insulin resistance in living subjects and its implication on cognitive impairment/ AD is difficult to establish due to inaccessibility of brain tissue. In this study we isolated and characterized plasma neuron‐derived small extracellular vesicles (NDE), and adopted multi‐omics approaches to discover novel biomarkers of AD and CNS insulin resistance and suggested their possible association.

**Method:**

Plasma samples were obtained from Look AHEAD‐MIND study with 28 cognitively normal (18 female and 10 male) and 28 individuals with probable dementia (11 female and 17 male) based on central adjudication by an expert panel. These individuals had established type 2 diabetes (T2D) and overweight/obesity when enrolled in a randomized controlled clinical trial of a 10‐year behavioral intervention. NDE were enriched from plasma sequentially using CD171 and synaptophysin surface markers by immunoprecipitation and characterized for non‐coding RNAs by small RNA‐sequencing and proteomics by mass spectrometry.

**Result:**

The small RNA‐sequencing analysis identified the expression of over 100 miRNAs in NDE from both groups. The Comparison of differentially expressed miRNAs revealed 42 miRNAs to be downregulated, while 23 miRNAs were upregulated in individuals with dementia. Importantly, all these miRNAs (both up‐ and –downregulated) were found to be over‐represented in AD‐ and insulin signaling‐related pathways. Also, the targets of these miRNAs were associated with pathways like protein phosphorylation, autophagy, cell cycle and nervous system development. Alongside, the mass spectrometry‐based proteomics analysis identified 3 proteins (haptoglobin, complement component C6, and alpha‐2‐macroglobulin) to be significantly downregulated and 1 protein (desmocolin‐1) to be upregulated in the NDE of individuals with dementia. Interestingly, NDE showed upregulated expression of miR‐185‐5p, with concomitant downregulation of one of its potential targets complement protein C6, both known to be associated with AD and insulin signaling.

**Conclusion:**

The study demonstrated multi‐omics approach to identify novel plasma NDE biomarkers for dementia pathogenesis and its association with CNS insulin resistance in individuals with T2D.

